# A Mathematical Model of CR3/TLR2 Crosstalk in the Context of *Francisella tularensis* Infection

**DOI:** 10.1371/journal.pcbi.1002757

**Published:** 2012-11-01

**Authors:** Rachel Leander, Shipan Dai, Larry S. Schlesinger, Avner Friedman

**Affiliations:** 1 Mathematical Biosciences Institute, The Ohio State University, Columbus, Ohio, United States of America; 2 Center for Microbial Interface Biology, Department of Microbial Infection and Immunity, The Ohio State University, Columbus, Ohio, United States of America; Utrecht University, Netherlands

## Abstract

Complement Receptor 3 (CR3) and Toll-like Receptor 2 (TLR2) are pattern recognition receptors expressed on the surface of human macrophages. Although these receptors are essential components for recognition by the innate immune system, pathogen coordinated crosstalk between them can suppress the production of protective cytokines and promote infection. Recognition of the virulent Schu S4 strain of the intracellular pathogen *Francisella tularensis* by host macrophages involves CR3/TLR2 crosstalk. Although experimental data provide evidence that Lyn kinase and PI3K are essential components of the CR3 pathway that influences TLR2 activity, additional responsible upstream signaling components remain unknown. In this paper we construct a mathematical model of CR3 and TLR2 signaling in response to *F. tularensis*. After demonstrating that the model is consistent with experimental results we perform numerical simulations to evaluate the contributions that Akt and Ras-GAP make to ERK inhibition. The model confirms that phagocytosis-associated changes in the composition of the cell membrane can inhibit ERK activity and predicts that Akt and Ras-GAP synergize to inhibit ERK.

## Author Summary

In the current work we construct a highly contextual model of membrane-proximal crosstalk between the ERK and PI3K cascades that is initiated through contact with *F. tularensis*. The model is used to test the hypothesis that phagocytic signaling downstream from CR3 is responsible for an early inhibition of ERK activity, which is seen subsequent to contact with the complement C3-opsonized Schu S4 strain of *F. tularensis*. In addition, the model predicts that Akt and Ras-GAP synergize to inhibit ERK. To the best of our knowledge this is the first mathematical model to investigate crosstalk between these pathways within the context of infection. By providing a comprehensive picture of the initial host-pathogen interaction, and pathogen-induced crosstalk between cell surface receptors in particular, this model is important in the context of microbial immunopathogenesis.

## Introduction

Receptor-mediated engagement followed by phagocytosis by professional phagocytes is the first critical step in microbial clearance or, in the case of intracellular pathogens, entry to a safe niche. The molecular mechanisms underlying phagocytosis are complex, usually involving more than one receptor and rapidly culminating in the combinatorial generation of a variety of biochemical signals along with rearrangement of the actin cytoskeleton to engulf the microbe [1]. There are substantial differences in cellular responses for almost every phagocytic receptor used, and complex interactions between receptors can be expected since a variety of ligands usually coat microbes. In this context, computational modeling becomes an essential tool through which experimentalists can enhance their understanding.

Complement Receptor 3 (CR3; CD11b/CD18), the major 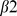 integrin of phagocytic cells (monocytes, macrophages and neutrophils), provides a highly effective mode of entry for many microbes and has long been postulated to provide the microbe safe passage into macrophages in particular, since ligation of CR3 by complement-opsonized microbes does not uniformly trigger toxic host cell responses [2]. Many intracellular pathogens use CR3 to evade intracellular killing [3]–[9]. Still, CR3 is a notoriously enigmatic receptor, capable of conveying diverse and even opposing signals in response to distinct combinations of ligands [10]–[13] and often in concert with pattern recognition receptors (PRRs) such as Toll-like Receptors (TLRs). A mounting body of research suggests that 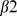 integrins are important regulators of TLR signaling [14]–[17]. The mechanisms by which CR3 regulates TLR signaling are an area of active research, in part because CR3/TLR crosstalk is implicated in the pathogenesis of several diseases.

*Francisella tularensis* is an extremely virulent intracellular pathogen of macrophages and potential bioweapon. Indeed, the bacteria may be aerosolized and inhalation of as few as ten bacteria can result in the fatal disease pneumonic tularemia [18]–[20]. In the lung, *F. tularensis* is rapidly phagocytosed by alveolar macrophages while suppressing their cytokine production. One mechanism the bacterium uses to accomplish this feat is to selectively engage only a few choice receptors. Although multiple types of receptors can mediate phagocytosis of *Francisella*, appreciable phagocytosis of the most virulent strains requires CR3 engagement by complement C3-opsonized bacteria [19], [21], [22]. In fact, CR3 is thought to be critical to the success of *F. tularensis* as an intracellular pathogen [18], [19], [21], [23]–[26]. Cytokine production in response to *Francisella* comes almost exclusively from its stimulation of TLR2 [18], [19], [27]. As noted above, although TLR2 signaling is inflammatory, it is also subject to regulation by CR3 [15], [17].

In what follows we construct a model of immediate membrane proximal signaling in response to *F. tularensis*. The model, which serves as a formal hypothesis, is shown to be consistent with the experimental results of S. Dai et al (unpublished data). Its implications are explored via numerical simulations.

## Results

The response of macrophages to *F. tularensis* depends heavily on the presence of complement. Dai et al found that complement opsonization substantially decreases cytokine production in response to *F. tularensis*, and identified key players in this immuno suppressive pathway (unpublished data). Their results are summarized as follows: ERK activation in response to *F. tularensis* is suppressed by complement-mediated signaling through CR3. Furthermore, ERK inhibition is rapid, being evident just 5 minutes post infection. In addition to suppressing ERK activation, CR3 ligation induces the rapid activation of Lyn kinase, which functions to inhibit cytokine production in response to *F. tularensis*. Finally, TLR2 and CR3 signaling intersect at the PI3K/Akt pathway, and the two receptors cooperate to support a complement dependent enhancement of Akt activity in response to *F. tularensis*.

The observations of Dai et al were supplemented with existing literature to construct a model of the very earliest signaling events that occur in response to *F. tularensis* infection. In this model TLR2-induced ERK activation occurs through a previously characterized MyD88 independent pathway in which Rac and Ras associate with the cytoplasmic domain of TLR2 and undergo rapid activation in response to bacterial stimuli [28], [29]. The pair then cooperate to activate Raf which leads to ERK activation [30]. Activation of the PI3K/Akt pathway by TLR2, meanwhile, is mediated by Rac [29]. In our model of complement-mediated signaling, CR3 ligation leads to the rapid activation of Lyn which subsequently activates PI3K [31], [32]. PI3K activation leads to a buildup of PtdIns(3,4)P (abbreviated here as PI(34)P) and PtdIns(3,4,5)P (PI(345)P) at the phagosomal cup (consistent with Clemens et al [21]), which antagonizes ERK. Specifically, Akt, which is activated after binding to these lipids, phosphorylates Raf at Ser 259 thereby inhibiting its association with Ras [33], and these lipids recruit GAPs, which deactivate both Rac and Ras [34]–[37]. The model also includes additional interactions which may detract from its ability to explain complement-mediated ERK inhibition. In particular, PI(34)P and PI(345)P can also recruit the Rac-GEF, Vav, [38], which initiates Rac activation, and Lyn can enhance Raf signaling [39].

**Figure 1:**
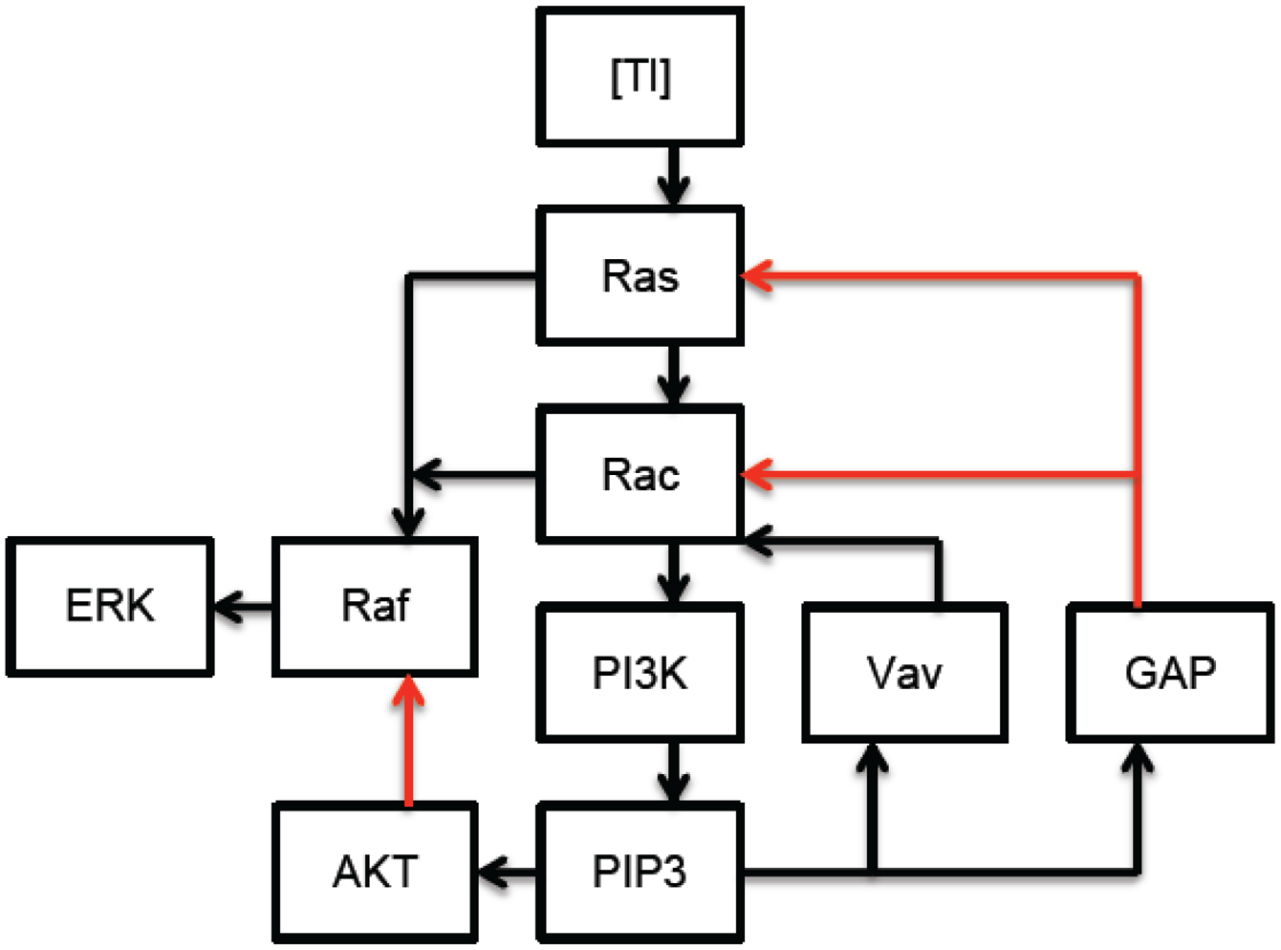
Membrane proximal Toll-like Receptor 2 signaling in response to *Francisella tularensis*: Ligand bound TLR2 (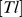) signaling leads to the sequential activation of Rac and Ras GTPases. Rac and Ras cooperate to activate Raf which leads to ERK activation, while Rac stimulates the PI3K/Akt pathway which antagonizes ERK by inhibiting Raf. The lipid product of PI3K, PIP3, also recruits positive (Vav) and negative (GAP) regulators of Ras and Rac to the immunological synapse. (Red arrows denote inhibition, black arrows denote stimulation.)

**Figure 2:**
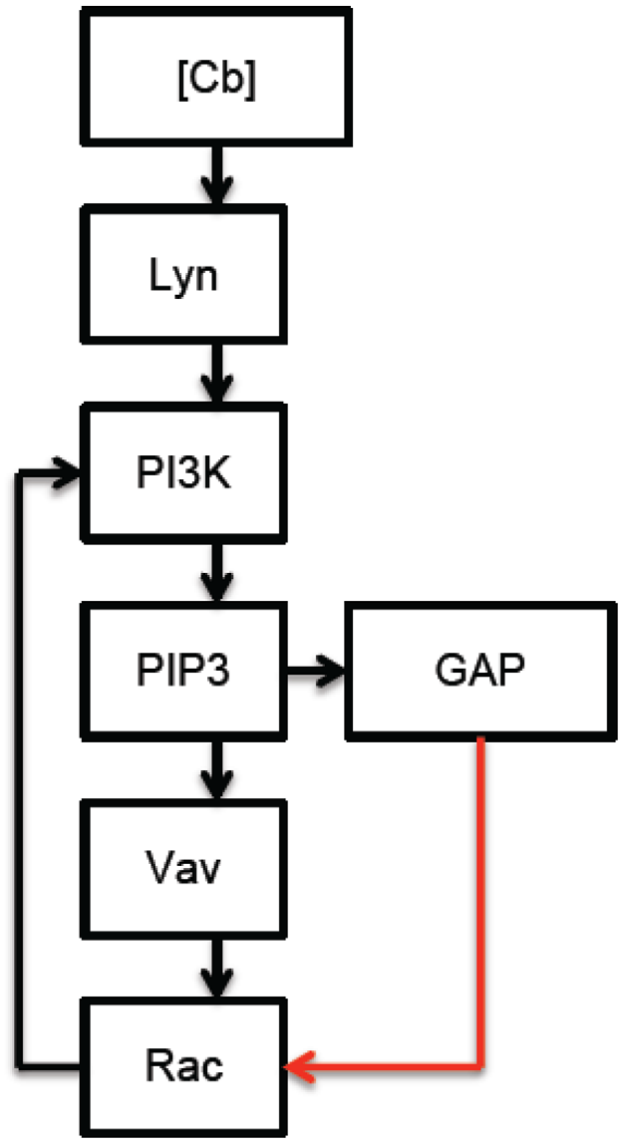
Membrane proximal Complement Receptor 3 signaling in response to complement C3-opsonized *Francisella tularensis*: Complement-bound CR3 (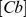) activates Lyn, which leads to the activation of PI3K and the accumulation of PIP3 at the immunological synapse. PIP3 recruits positive (Vav) and negative (GAP) regulators of Rac, while Rac contributes to PI3K stimulation. (Red arrows denote inhibition, black arrows denote stimulation.)

**Figure 3:**
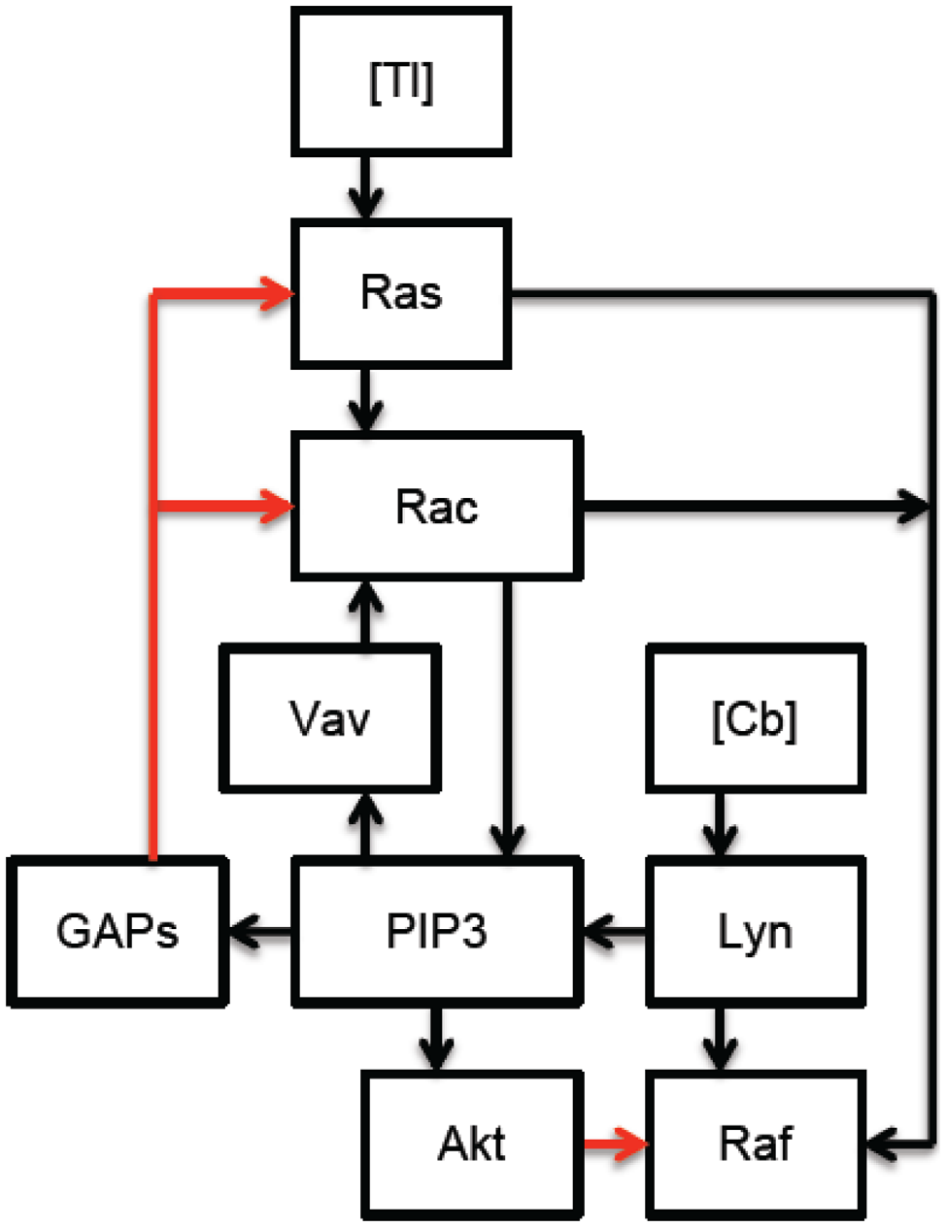
Membrane proximal CR3/TLR2 crosstalk in response to complement C3-opsonized *Francisella tularensis*: Ligand bound TLR2 (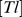) signaling leads to the sequential activation of Rac and Ras GTPases. Rac and Ras cooperate to activate Raf, while Rac stimulates the PI3K/Akt pathway. In addition, complement-bound CR3 (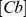) activates Lyn, which leads to the activation of PI3K and the accumulation of PIP3 at the immunological synapse. PIP3, recruits positive (Vav) and negative (GAP) regulators of Ras and Rac to the immunological synapse. (In this simplified model active Raf serves as a proxy for active ERK, as explained in the text. Red arrows denote inhibition, black arrows denote stimulation.)

Figures 1 and 2 provide a schematic description of membrane proximal TLR2 and CR3 signaling in response to *Francisella tularensis*. Figure 3 synthesizes and simplifies the CR3 and TLR2 signaling networks. In particular, in the interest of simplicity, our model uses the concentration of active Raf as a proxy for the concentration of active ERK. Although significant feedback from ERK to Raf could alter the model's dynamics, this simplification seems reasonable in view of the following facts: ERK mediated feedback is not significant until later time points [40], and the proposed mechanisms of CR3-mediated ERK inhibition target molecules that lie upstream of ERK itself.

Because the network is complex, we resort to mathematical modeling in order to deduce function from structure, that is, in order to check that the model is in fact consistent with experimental observations. The model equations, based on Figure 3, give the local concentrations of various signaling molecules in the vicinity of the immunological synapse. In these equations 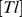 represents ligand bound TLR2 heterodimers; 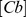 represents ligand bound CR3; 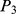 represents 3 phosphoinositides (i.e. both PI(345)P and PI(34)P); 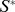 represents active Ras; 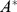 represents active Akt; 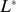 represents active Lyn; 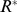 represents active Rac; and 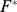 represents active Raf. The model equations are given in the section Materials and Methods where they are supplemented by Tables 1 and 2 of parameter values.

**Table 1:** Parameter values.

parameter	Value	reference
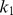	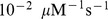	[52], Estimate
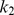	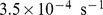	[52], Estimate
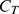	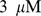	[58] (monocytes)
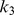	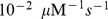	[60]
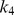	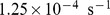	[60]
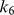	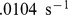	[96]
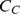	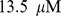	[62]
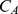	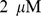	[69] (PC12)
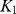	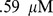	[68]
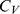	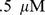	[107], Estimate
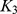	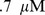	[79], Estimate
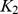	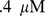	[34], [37], [108], Estimate
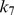	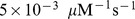	[63], [64] Estimate
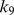	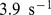	[87]
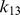	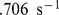	[83]
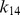	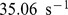	[84]
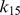	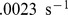	[83]
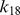	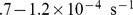	[83]
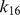	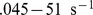	[104]–[106]
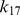	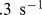	[101]–[103]
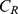	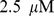	[82] (neutrophil)
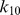	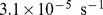	[86]
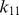	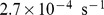	[109]
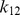	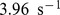	[88]
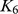	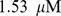	[88]
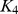	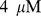	[37]
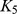	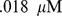	[100]
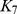	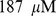	[83]
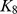	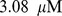	[84]
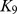	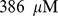	[87]
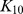	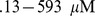	[104], [105]
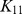	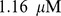	[101]–[103]
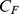	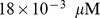	[69] (COS)
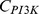	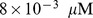	[69] (NIH3T3)
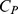	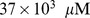	[110], [111]
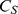	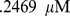	[85] (NIH3T3)
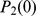	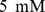	[97] (neutrophil)
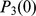	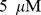	[97] (neutrophil)
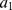	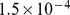	[49], [50]

Parameter values taken from the literature.

**Table 2:** Parameter values.

parameter	value
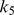 *	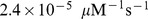
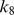	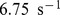
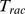	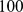
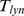	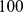
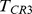	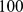
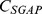	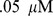
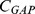	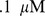
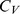	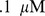
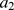	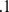

The baseline values of unknown parameters and estimated parameters.

* Estimated from initial conditions.

We next present the results of numerical simulations on the mathematical model. In order to test the model's consistency we compare the results of simulations in the presence and absence of complement.

**Figure 4:**
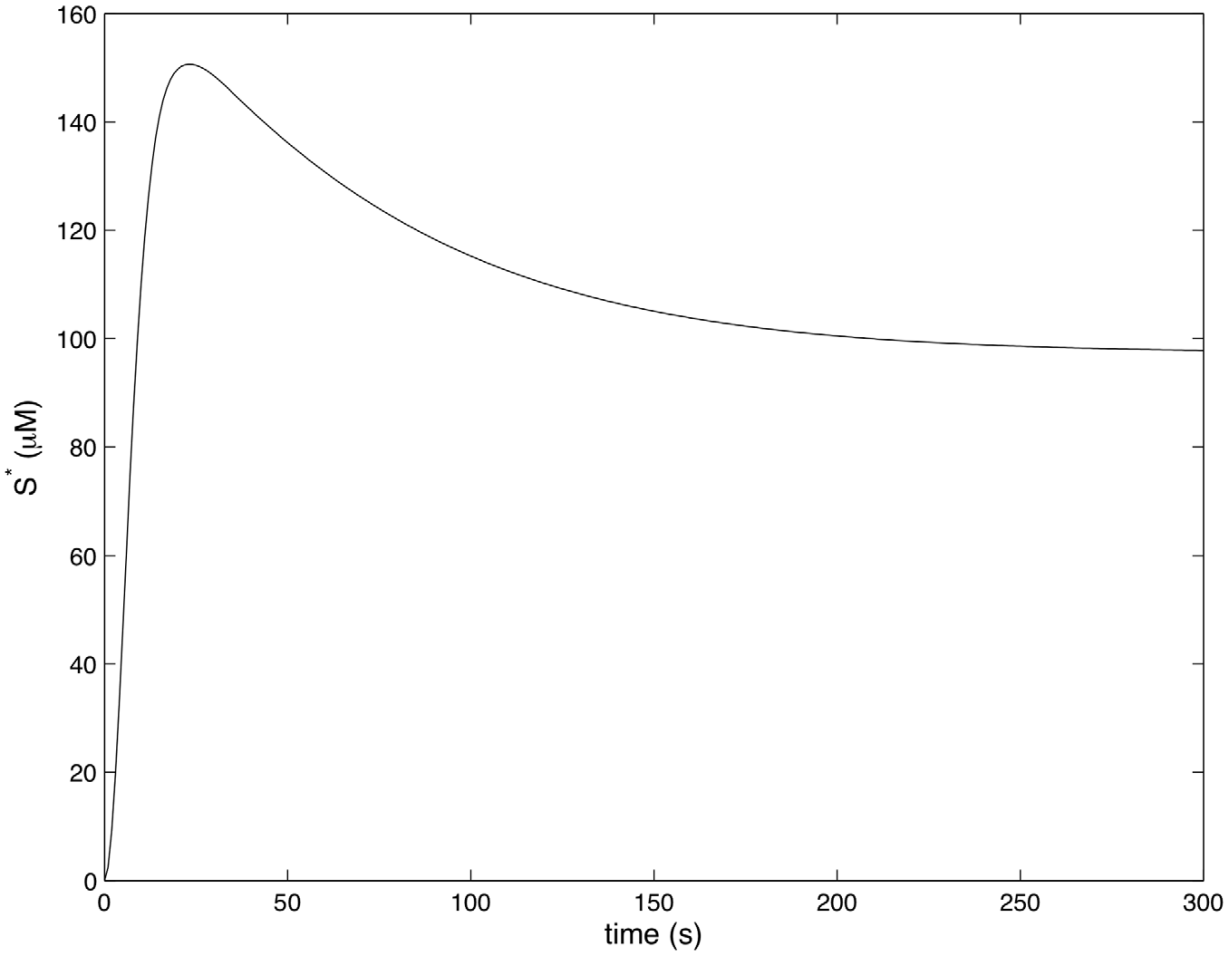
Ras activity in response to unopsonized *Francisella*. (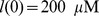, 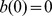).

**Figure 5:**
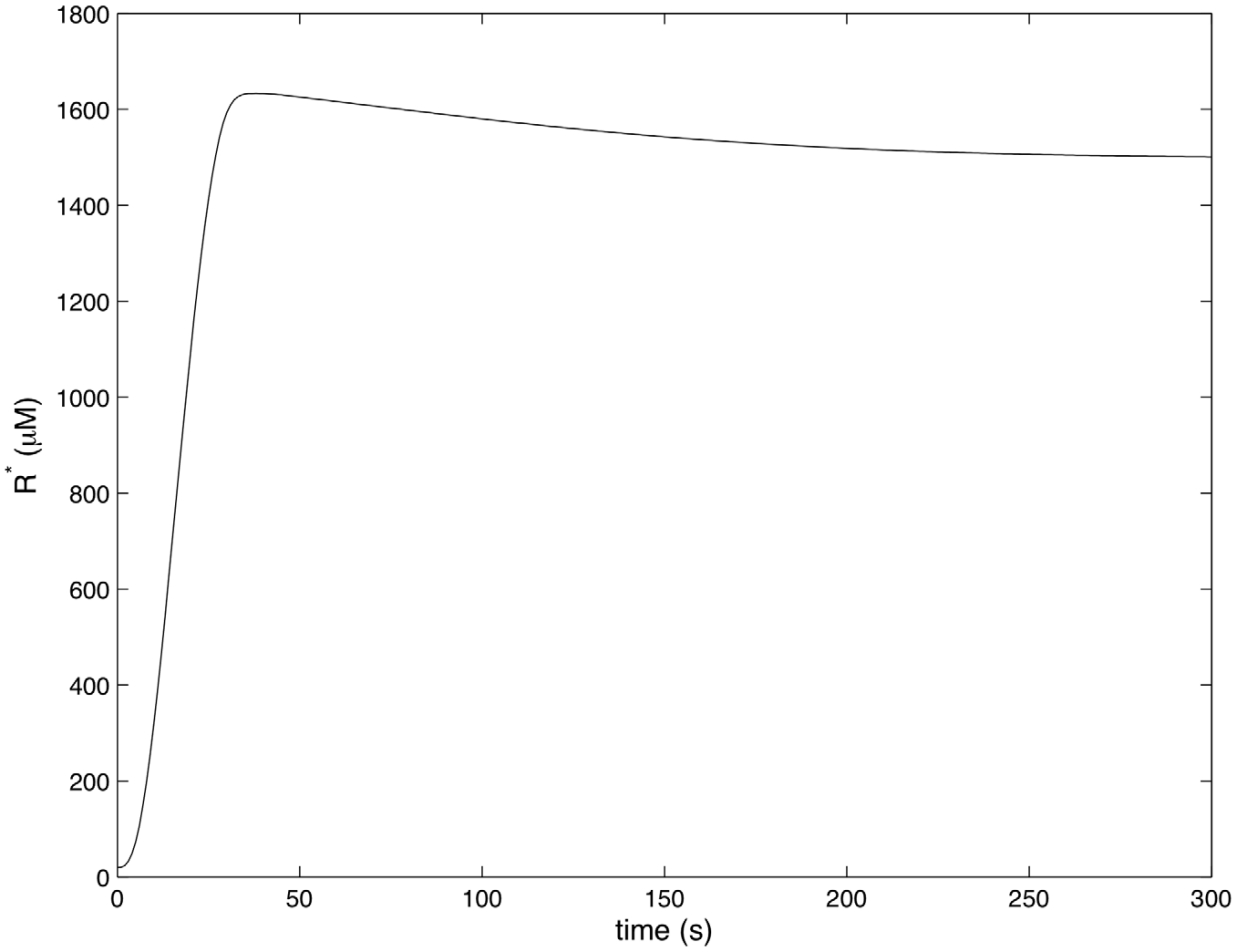
Rac activity in response to unopsonized *Francisella*. (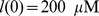, 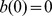).

**Figure 6:**
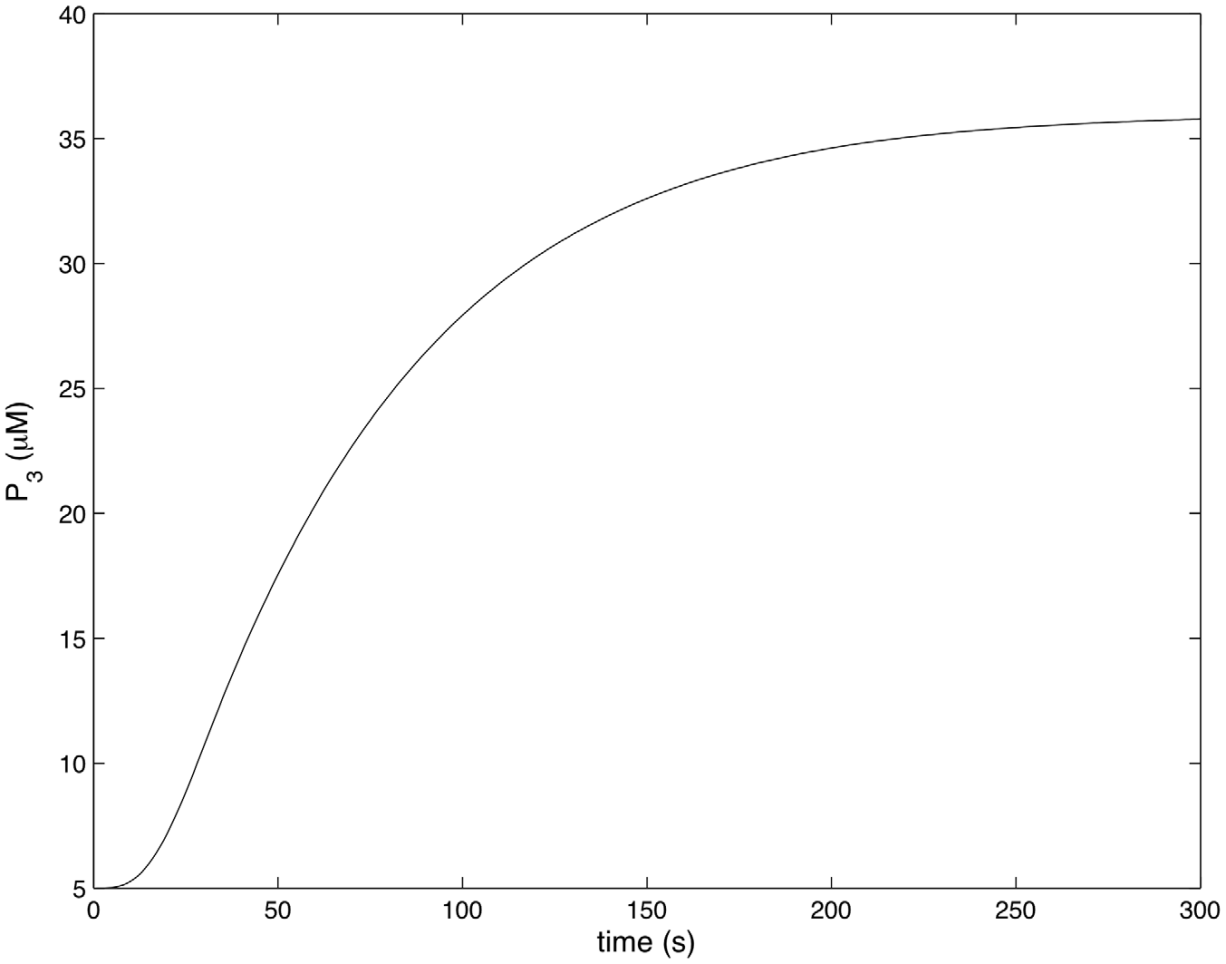
The concentration of 3 Phosphoinositides in response to unopsonozed *Francisella*. (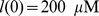, 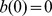).

**Figure 7:**
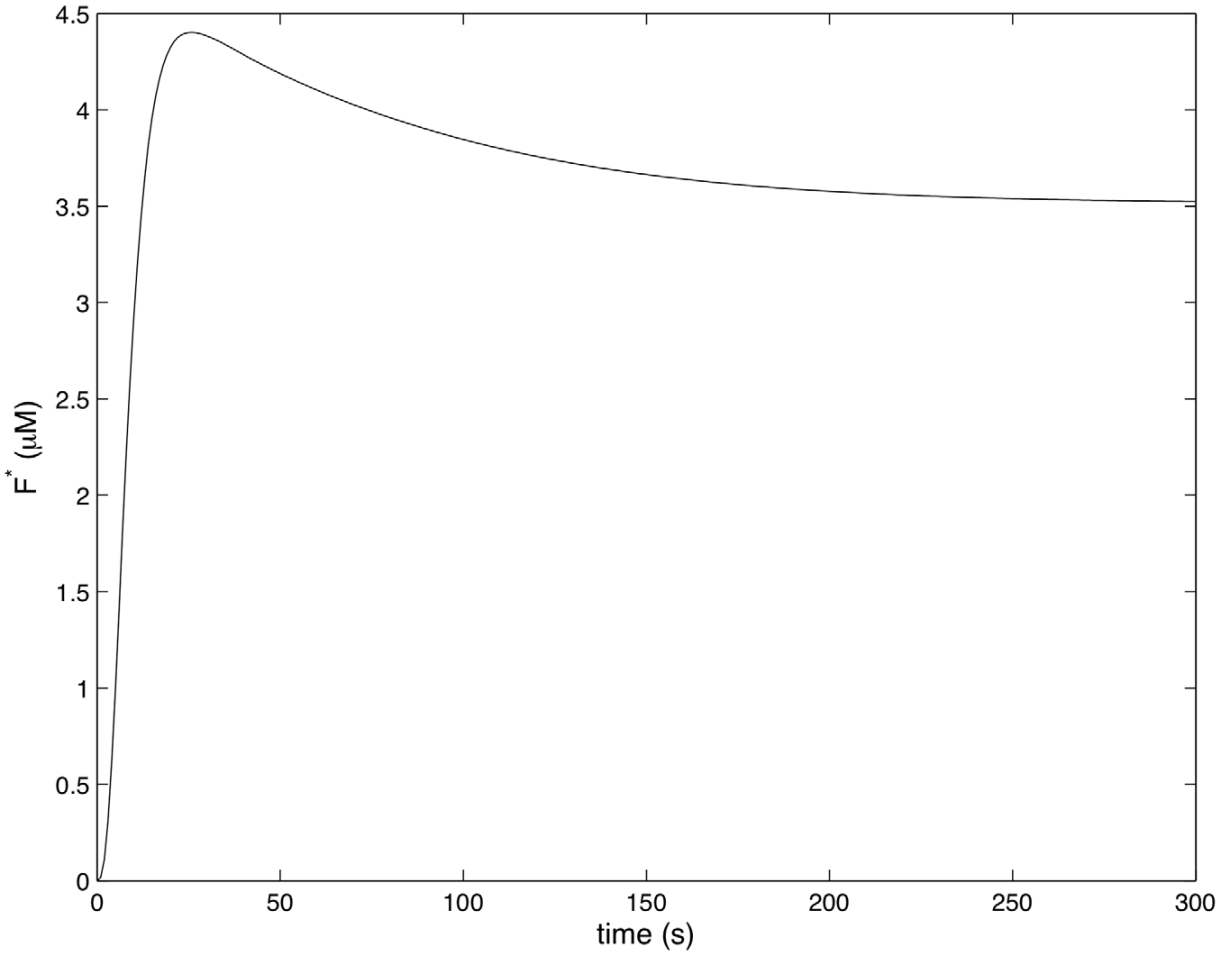
Raf activity in response to unopsonized *Francisella*. (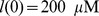, 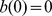).

Figures 4–7 show how the concentrations of signaling molecules change through time, when the bacteria are not opsonized, but carry a low density TLR2 ligand. In the absence of complement the model predicts that *Francisella* will elicit a slight increase in 3 phosphoinositides and a substantial increase in active Ras, Rac and Raf. As Raf is a proxy for ERK, we see that in the absence of complement the model is in agreement with the experimental results of S. Dai et al (unpublished data). In particular, TLR2 signaling stimulates both the ERK and PI3K pathways.

**Figure 8:**
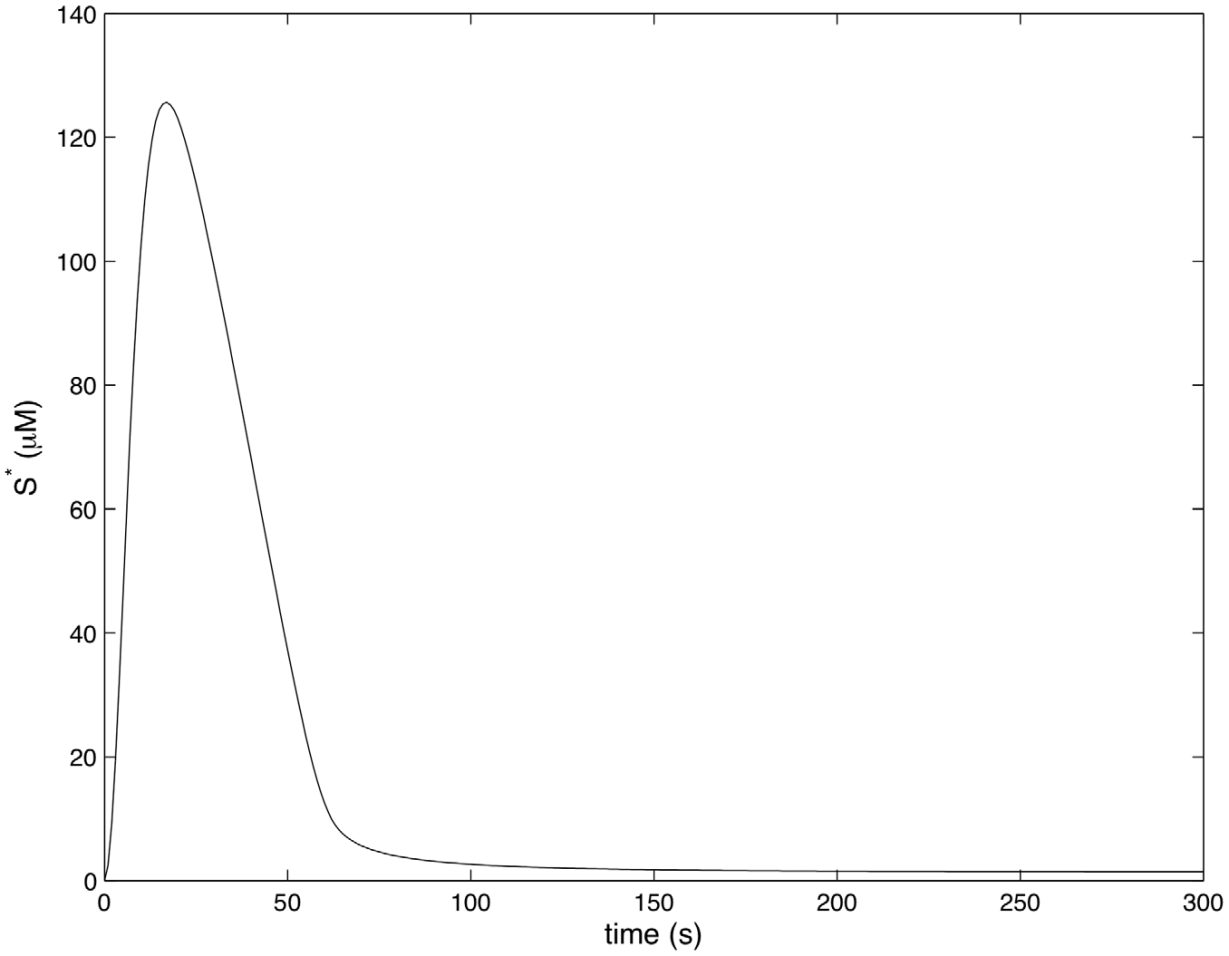
Ras activity in response to complement C3-opsonized *Francisella*. (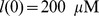, 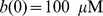).

**Figure 9:**
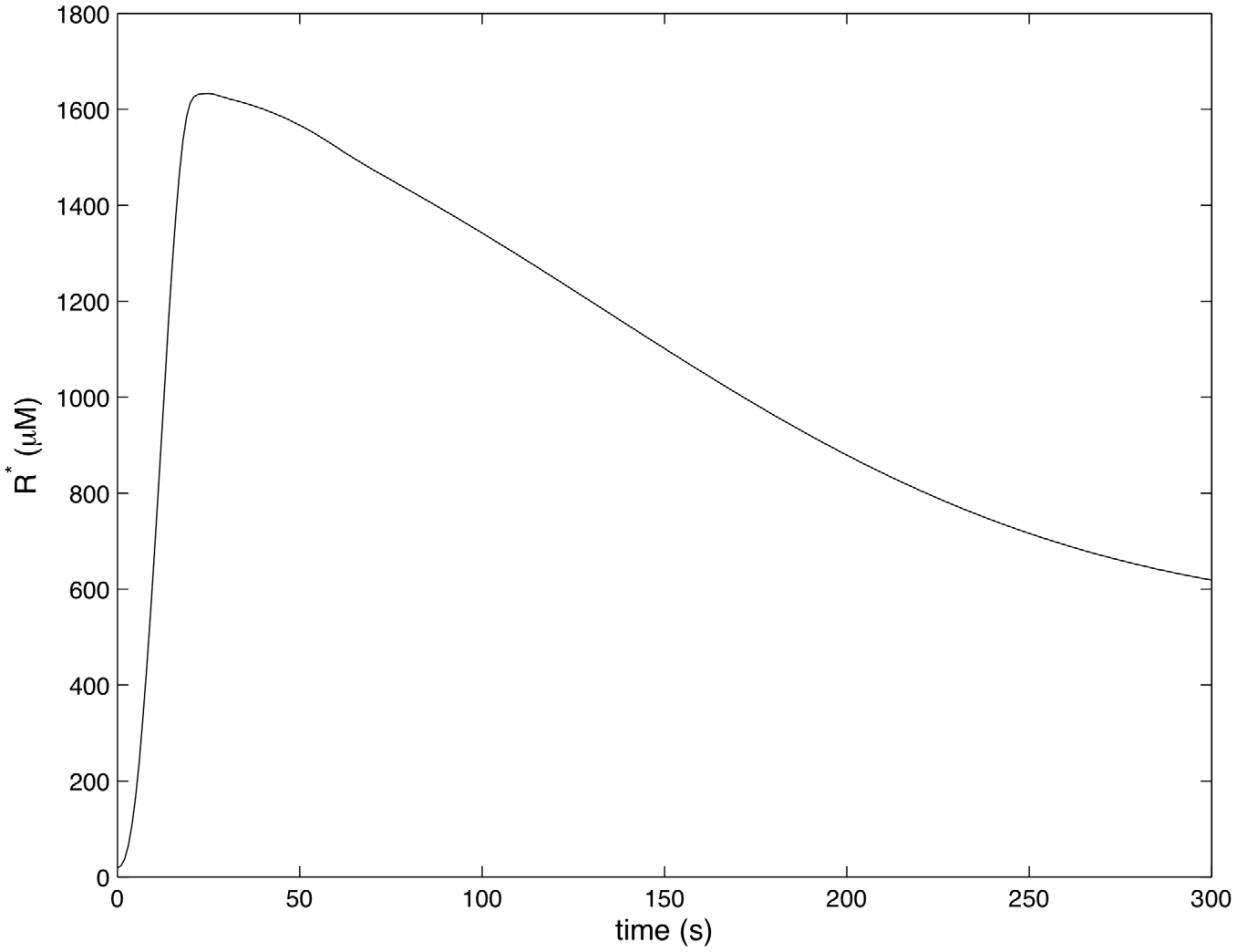
Rac activity in response to complement C3-opsonized *Francisella*. (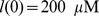, 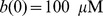).

**Figure 10:**
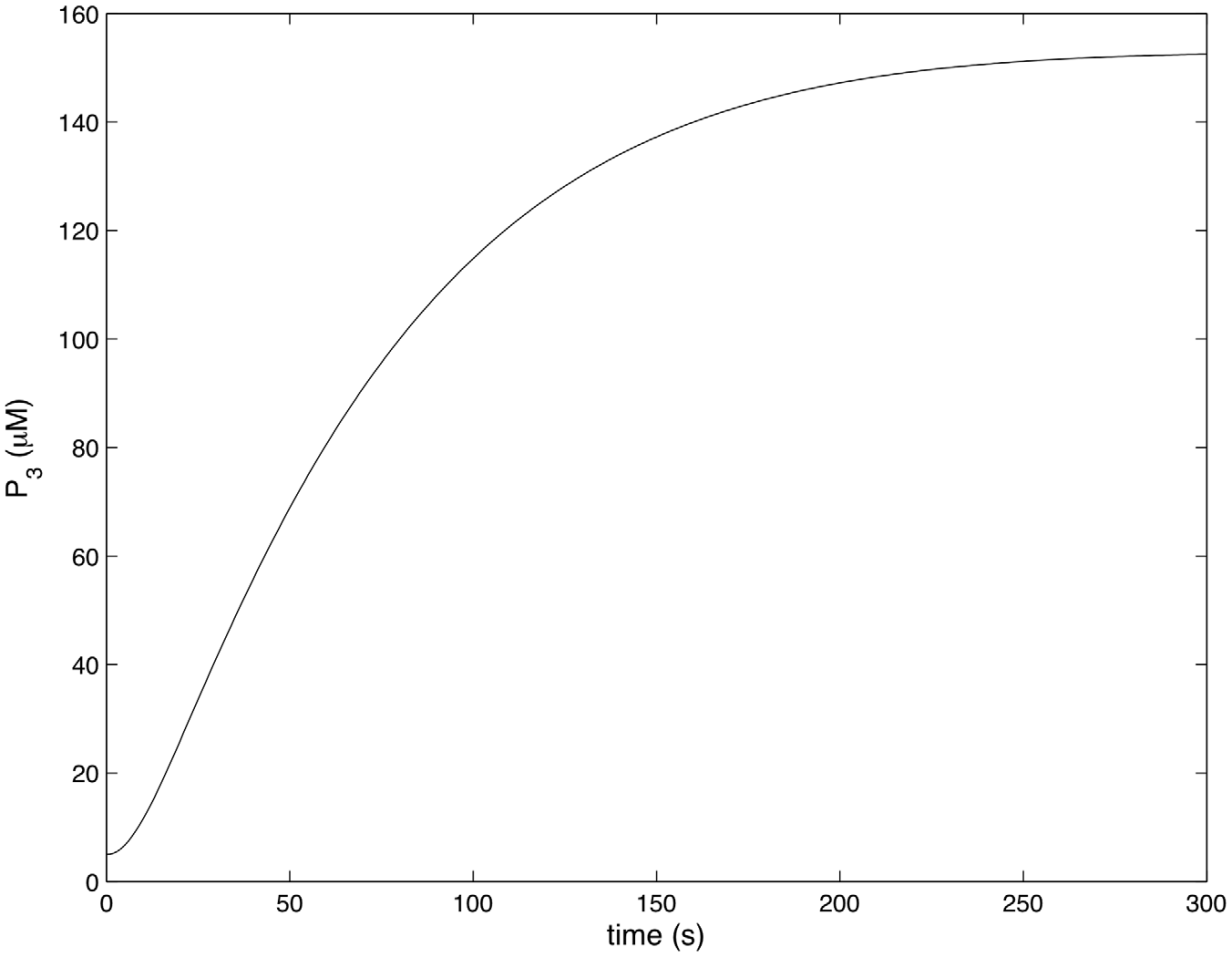
The concentration of 3 Phosphoinositides in response to complement C3-opsonozed *Francisella*. (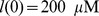, 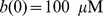).

**Figure 11:**
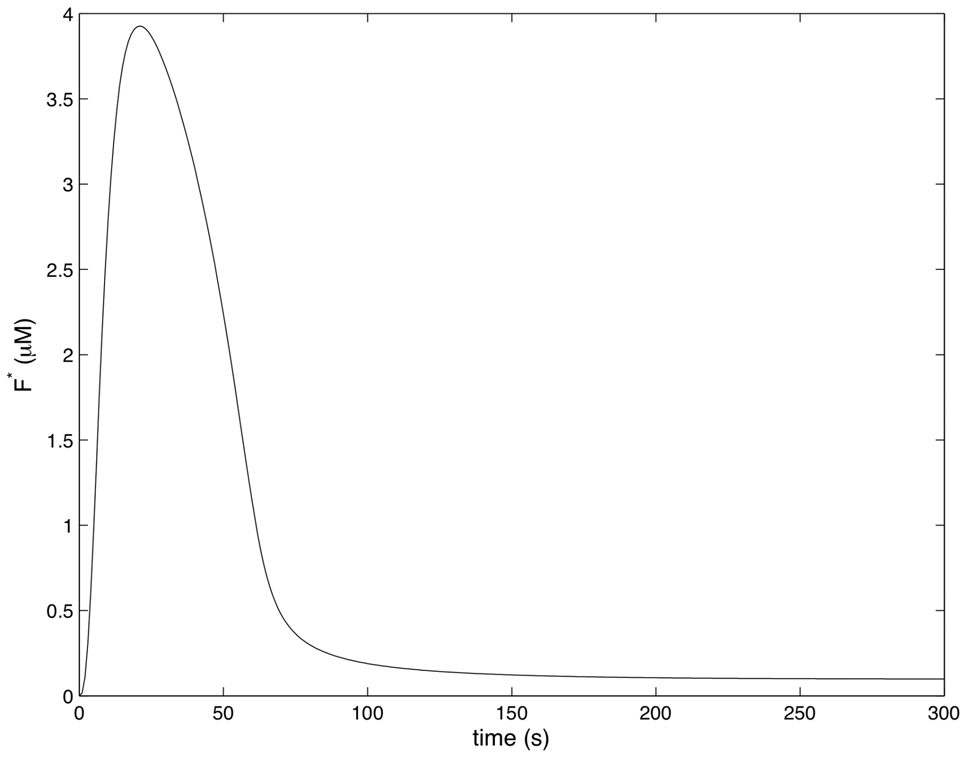
Raf activity in response to complement C3-opsonized *Francisella*. (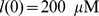, 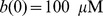).

Figures 8–11 show how the concentrations of the above signaling molecules change through time in response to opsonized *Francisella*. A comparison of Figures 7 and 11 shows that the model proposed in Figure 3 is consistent with the experimental data, and in particular, is capable of explaining CR3-mediated ERK inhibition. Specifically, Figure 11 shows that *F. tularensis* induced Raf stimulation is markedly inhibited in the presence of complement.

**Figure 12:**
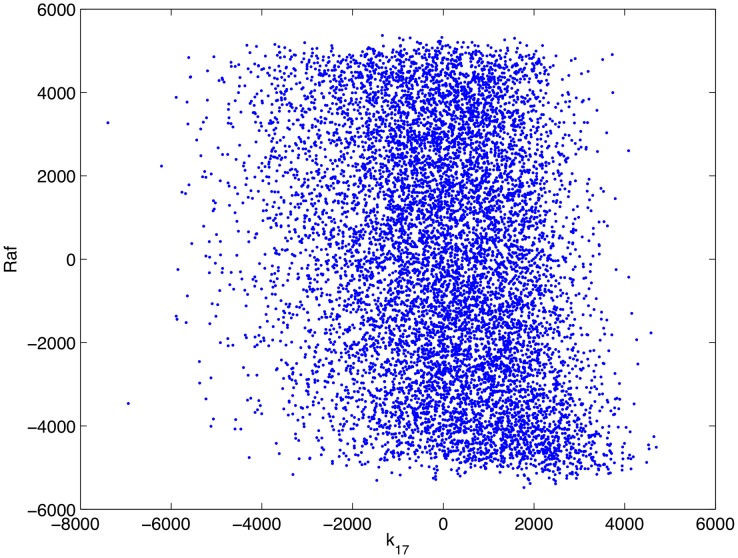
Scatter plot of rank transformed Raf concentration at 5 minutes post infection versus the rank transformed value of the parameter 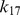.

**Figure 13:**
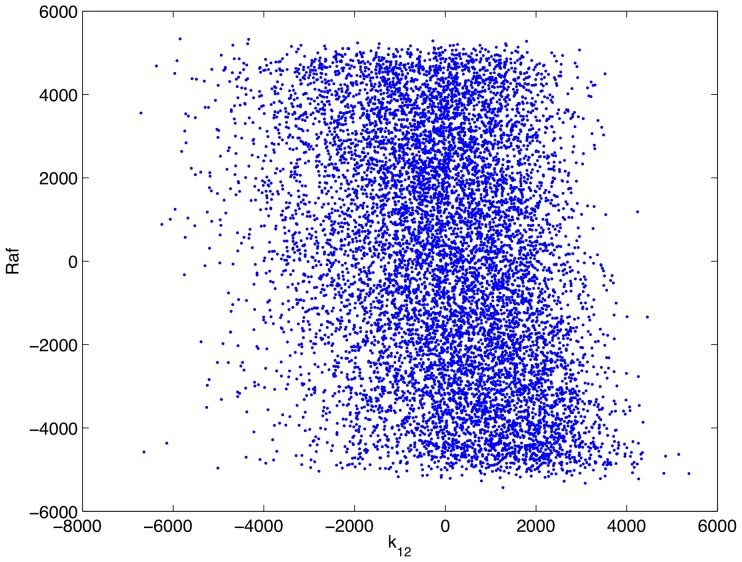
Scatter plot of rank transformed Raf concentration at 5 minutes post infection versus the rank transformed value of the parameter 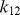.

**Figure 14:**
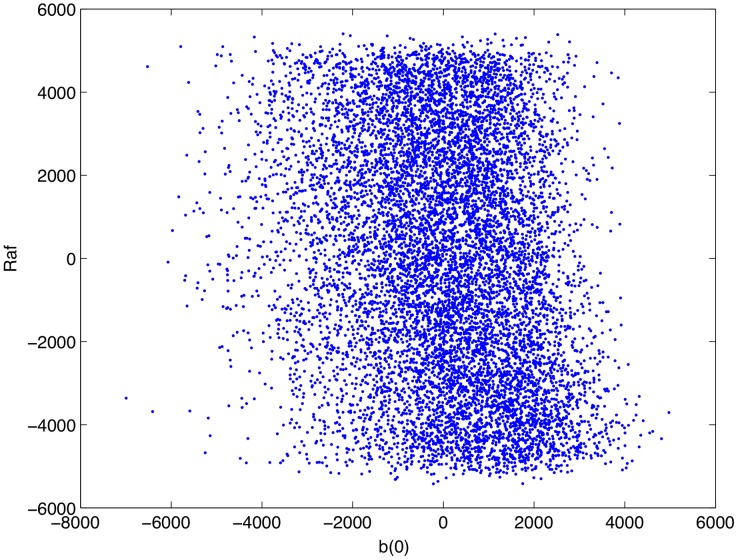
Scatter plot of rank transformed Raf concentration at 5 minutes post infection versus the rank transformed value of the parameter 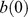.

**Figure 15:**
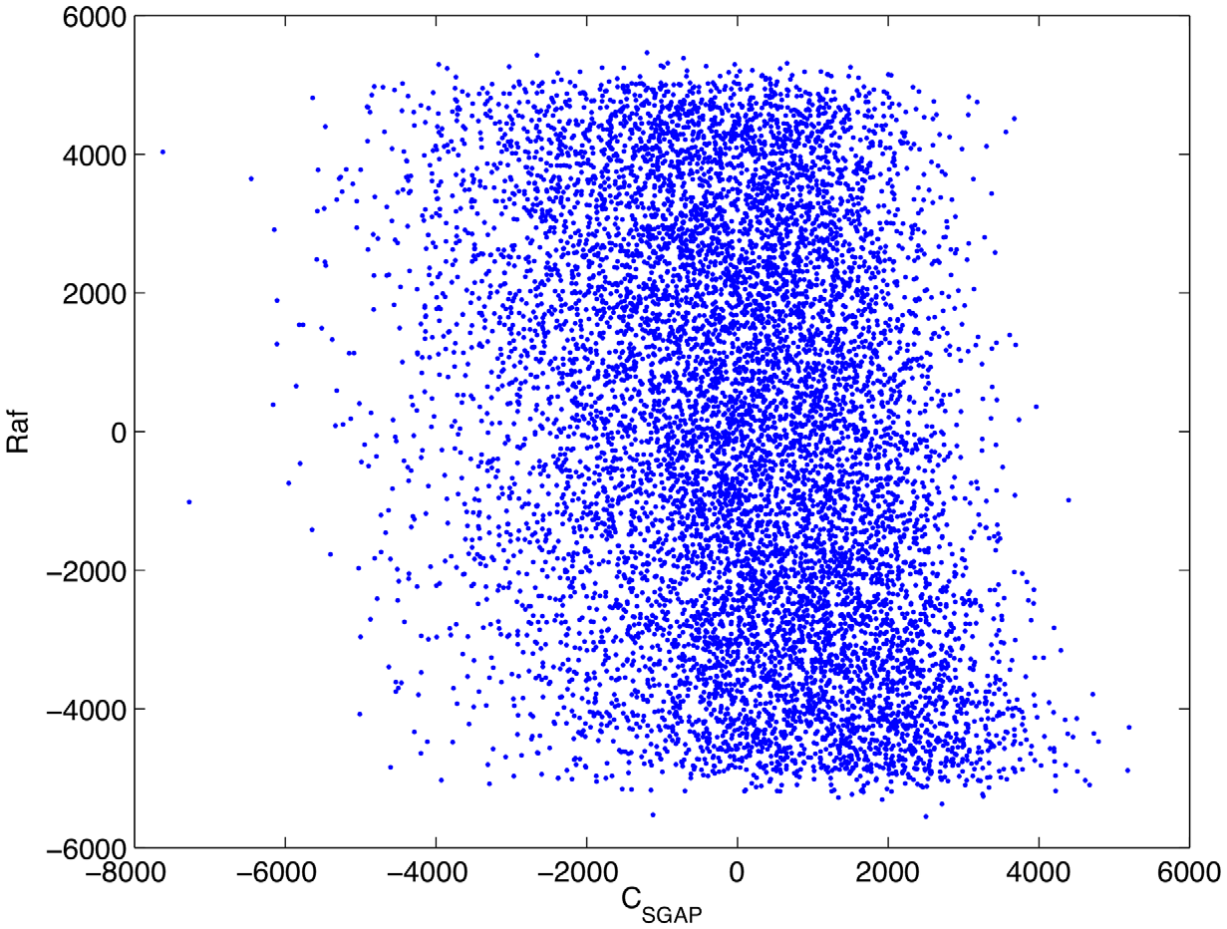
Scatter plot of rank transformed Raf concentration at 5 minutes post infection versus the rank transformed value of the parameter 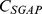.

Having confirmed that, as parameterized, the mathematical model is consistent with complement-mediated ERK inhibition we performed an uncertainty and sensitivity analysis in order to assess how uncertainty in the model's parameters impacts its consistency with experimental data. In particular, as some of the model's parameters are uncertain, we wished to know if complement-mediated ERK inhibition is robust to variations in the model's parameters, i.e. is the model consistent with experimental result over a wide range of parameter values. We ran 10,000 numerical simulations in which the model's parameters were varied according to a Latin hypercube sampling scheme. The sensitivity of the model's output (as measured by the concentration of active Raf at 5 minutes post infection) to uncertainty in the parameters was then quantified through a partial rank correlation coefficient [41] that is, we calculated the partial correlation coefficients of the rank transformed data. This provides a robust sensitivity measure of nonlinear but monotonic relations between the parameters and the output [41]. A detailed description of the process is presented in [41]. The results of this analysis are presented in Table 3. Figures 12–15 show scatter plots of rank transformed Raf concentration at 5 minutes versus rank transformed parameter values for a few of the most important parameters. Importantly, 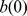, the concentration of complement on the surface of the bacteria shows a significant negative correlation with the concentration of active Raf, i.e. the model is consistent with experimental data over a wide range of parameter values.

**Table 3:** PRCC values.

parameter	range	PRCC value	parameter	range	PRCC value
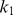	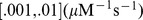	.0209	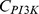	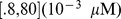	.0004
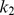	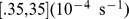	−.0006	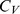	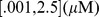	.1722*
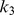	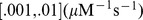	−.0160	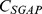	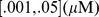	−.3042*
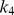	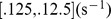	.0129	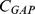	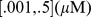	−.1336*
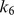	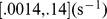	−.0187	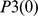	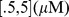	−.3005*
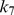	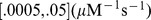	.0142	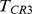	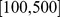	−.0080
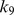	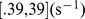	.3629*	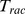	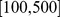	−.0671*
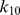	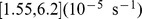	−.0118	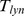	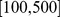	−.0608*
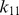	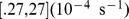	.0024	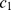	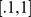	.0524*
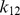	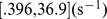	−.3150*	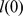	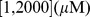	.0432*
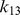	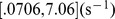	.1904*	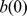	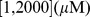	−.2498*
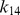	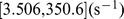	−.1326*	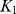	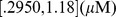	.0010
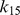	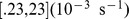	−.0285*	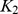	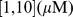	.1226*
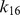	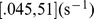	.3269*	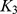	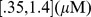	−.0211
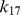	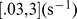	−.2482*	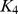	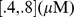	.1480*
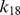	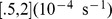	−.0039	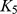	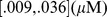	−.0909*
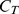	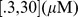	.3433*	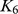	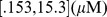	.1303*
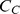	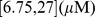	.1169*	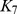	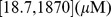	−.0226
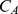	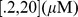	−.0133	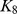	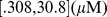	.0642*
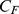	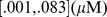	.6772*	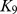	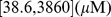	−.1980*
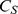	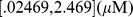	.3073*	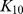	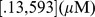	−.1080*
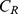	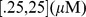	.0655*	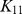	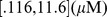	.0202

PRCC values for 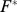 at 5 minutes post infection.

* denotes significant PRCC values (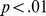).

In addition to showing that the model's consistency is robust to parameter variations, the uncertainty and sensitivity analysis can be used to identify molecules and parameters that are important for complement-mediated ERK inhibition. Although many of the parameters show a small but significant correlation with the concentration of Raf at 5 minutes post infection, the strongest negative correlations are associated with the parameters for GAP-mediated Ras deactivation, namely 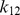 and 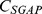, and 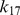, the rate of Akt catalyzed Raf phosphorylation. This suggests that both Ras-GAPs and Akt are important regulators of Raf. To analyze the relative importance of Ras-GAPs and Akt in CR3-mediated Raf inhibition, we ran numerical experiments in which the 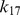 and 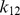 were reduced to 10% of their baseline values, and the activity of Raf 5 minutes post infection with opsonized *F.tularensis* was compared to that when the parameters were set to their baseline values. A ten percent reduction in 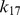 resulted in a 8.5 fold increase in the concentration of Raf at five minutes post infection, whereas a ten percent reduction in 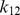 resulted in a 5.5 fold increase in Raf activation 5 minutes post infection. When both parameters were reduced to 10% of their baseline values, the model predicts a 31.5 fold increase in Raf activation at 5 minutes post infection, i.e. Akt and Ras-GAPs synergize to inhibit Raf signaling. The uncertainty and sensitivity analysis also indicates that CR3-mediated ERK inhibition is sensitive to concentrations of key cellular proteins. In particular, it indicates that over expression of TLR2 or Ras, or reduced expression of Ras-GAP will dampen CR3-mediated ERK inhibition.

## Discussion

Crosstalk between the complement and TLR systems is an essential determinant of the early immune response to pathogens [42]. In this paper we have presented a mathematical model of TLR2/CR3 crosstalk to test the hypothesis that CR3 ligation fosters the robust production of PI(34)P and PI(345)P which is incompatible with TLR2-mediated Raf signaling. Our own experimental data, in addition to the observation that less virulent strains of *Francisella* undergo PI3K-independent phagocytosis [43], lends support to the hypothesis that the membrane's phosphoinositide content during phagocytosis is a critical determinant of cytokine production in response to infection.

In defining crosstalk between CR3 and TLR2 within the context of *Francisella* infection, we also defined crosstalk between the ERK and PI3K signaling cascades within this context. Although several computational analyses of differential ERK signaling and ERK/PI3K crosstalk have already been performed [44]–[46], to the best of our knowledge this is the first mathematical model to investigate crosstalk between these two pathways within the context of infection. Although our model is by necessity a simplification it contains information about all of the major and immediate components in the CR3/phagocytic and TLR2/cytokine signaling pathways. In the case of CR3 signaling these components are Src family kinases, Rho family GTPases and lipids. In the case of TLR2 these components are Ras GTPase and Raf and Rac GTPase, Akt and lipids. By tracking the dynamics of these important and well characterized signaling molecules we confirm that the model reproduces experimental results, characterizes mechanisms of inhibition, and identifies targets for experimental manipulation. In particular, the model confirms that phagocytosis-associated changes in the composition of the cell membrane can inhibit ERK activity, predicts that Akt and Ras-GAP signaling synergize to inhibit ERK, and identifies Ras-GAP and Akt as a future target for experimental manipulation.

According to our model, CR3-mediated inhibition of TLR2 signaling initiated by *Francisella* is different from CR3-mediated inhibition of TLR2 signaling initiated by *Porphyromonas gingivalis*, wherein IL-12 production is inhibited by ERK [42]. This difference may stem from the fact that *P. gingivalis* binds to CR3 via its natural fimbriae, while *Francisella* cannot efficiently bind CR3 unless opsonized with C3bi. Or, it could also be that *P. gingivalis* engages other receptors that augment or interfere with CR3/TLR2 crosstalk. However, complement receptor-mediated PI3K activation has been observed to inhibit TLR-induced IL-12 production in response to Hepatitis C virus [47]. Furthermore, as C3bi ligation of CR3 is known to inhibit IL-12 production in response to a variety of stimuli [48], and support the pathogenesis of a variety of diseases [3]–[9], it seems likely that the model proposed here is applicable to a variety of pathogens.

## Materials and Methods

In this section we briefly describe the mechanisms through which the molecules of our model are regulated. Each verbal description is followed by a mathematical one. When coupled together, these mathematical descriptions form a model of membrane proximal receptor signaling in response to *F. tularesis*. Recruitment, the process through which diverse proteins translocate to a common location, is a central aspect of receptor signaling. Indeed, receptor-mediated recruitment concentrates activators, enzymes and substrates to specific locations on the inner leaflet of the cell membrane. In order to model recruitment we allow local concentrations of molecules at the immunological synapse to far exceed membrane and cellular concentrations. For example, receptors may be recruited to the immunological synapse through their ability to bind bacterial ligands. In this case, the maximal concentration of receptors at the synapse is bounded by 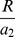, where 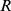 is the concentration of the receptor in the membrane, and 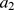 is the ratio of the volume of the synapse to the volume of the membrane. In this paper the term synapse loosely refers to the region of contact between bacteria and macrophage. In the context of the experiments that we seek to describe the multiplicity of infection is necessarily high, and so we take 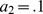. Similarly, when a cytoplasmic protein is recruited to the synapse, the maximal concentration of the protein at the synapse is given by 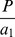, where 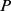 is the concentration of the protein in the cell and 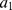 is the ratio of the volume of the synapse to the volume of the cell. Assuming that the macrophage is approximately spherical, that its membrane proximal region has a depth of 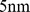 [49], and that an alveolar macrophage has a total diameter of 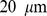 [50], we find that 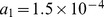.

### TLR2

TLRs are pattern recognition receptors (PRRs) that detect and respond to a broad range of pathogen products including bacterial lipoproteins [27], [51]. The molecular mechanisms that enable TLRs to respond to bacterial ligands are extremely complex, and can involve crosslinking of TLR heterodimers as well as multiple accessory proteins [52]. In particular, the recognition of pathogen associated molecular patterns (PAMPs) by TLR2, the most promiscuous of all the TLRs, is an involved process [53]. TLR2 is expressed on the cell surface where it constitutively associates with either TLR1 or TLR6 [54], [55]. TLR1 mediates the recognition of triacylated lipoproteins while TLR6 mediates the recognition of diacylated lipoproteins. We assume that the primary source of TLR1/2 stimulation is through direct contact with the bacterial membrane. Indeed TLR2 is recruited to developing phagosomes [56]. Ligand binding to TLR2 and subsequently TLR1 or TLR6 then induces a crosslinking of these receptors that initiates signal transduction [55], [57]. The diagram is as follows:
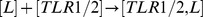
We denote by 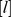 the total concentration of TLR1 ligand in the immunological synapse, and by 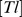 the concentration of the active signaling complex in the immunological synapse. Our quantitative description of 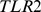 activation is as follows:
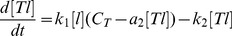
(1)

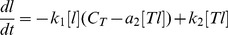
(2)

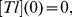
(3)where 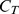 denotes the membrane proximal concentration of TLR1/2 heterodimers, which we assume is a constant.

In [52] it was shown that TLR2 binds lipoproteins directly with a 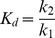 of 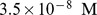. Assuming that the rate at which TLR2-lipoprotein complexes form, 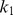, is approximately equal to the rate at which CR3-C3bi complexes form, i.e. 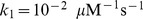, we estimate 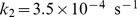.

According to [58] human monocytes have an average of 2100 molecules of TLR1 per cell. Assuming that monocytes are spherical, have a radius of 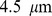, and that all of the TLR1 is concentrated in a 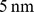 space around the membrane we estimate the membrane proximal concentration of TLR1/2 heterodimers is approximately 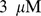. Under normal conditions, macrophages likely express lower levels of TLR1/2 than do monocytes [59].

### CR3 and Lyn

CR3 has an active conformation that binds to and mediates the phagocytosis of C3bi-opsonized particles and an inactive conformation that does not [32], [60]. As a result, inside-out signaling through receptors such as TLR2 is often viewed as important for CR3-mediated phagocytosis [15]. In macrophages, however, CR3 readily binds to C3bi-opsonized particles [61]. As a result, the following equations will be used to determine the concentration of CR3-C3bi complexes in the immunological synapse (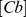).
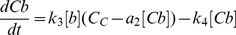
(4)

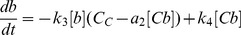
(5)

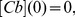
(6)where 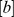 denotes the concentration of C3bi in the synapse and 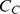 denotes the membrane proximal concentration of CR3, which we assume is a constant.

Cai et al [60] used a soluble monomeric probe, C3bi-AP, in order to estimate the on rate, 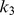, and the equilibrium dissociation constant, 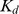, of C3bi for active CR3. We used 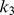 and 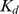 to calculate 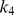. In vivo C3bi-CR3 complexes likely dissociate more quickly due to uncharacterized active cellular processes.

Ross et al [62] used labeled CR3 specific mAbs to determine the molecules of CR3 per alveolar macrophage. Assuming the previously described dimensions, we determined the membrane proximal concentration of CR3, 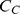.

Src family kinases, of which Lyn is a member, are rapidly activated in response to integrin ligation [32]. Although the precise mechanisms of integrin-mediated src kinase activation are unknown, some studies support a model in which inactive integrins associate constitutively with Src kinases which are then activated through trans-phosphorylation as a result of integrin clustering [32]. For simplicity we will assume that each molecule of CR3 is associated with a single molecule of Lyn. Under this assumption (7)–(8) determines the concentration of active Lyn (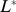).
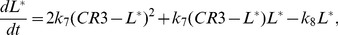
(7)

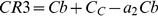
(8)where the first term is the rate of Lyn activation through the juxtaposition of two inactive CR3-associated Lyn molecules, and the second term is the rate of Lyn activation due to the juxtaposition of one active CR3-associated Lyn molecule and one inactive CR3-associated Lyn molecule.

Although we observe rapid activation of Lyn in response to *Francisella*, the precise rates of Lyn activation and deactivation within this context are not known. Several in vitro studies, however, enable us to estimate 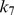, the rate of trans-phosphorylation [63], [64]. The rate of dephosphorylation is then chosen to ensure that in resting cells the concentration of phospho-Lyn is low [65]. Since the basal concentration of phospho-Lyn is inversely related to the ratio 
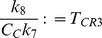
, we assume that 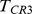 is large so that the basal concentration of phosph-Lyn is small.

### Akt

The activity of Akt is regulated by multiple kinases, phosphatases and lipids. In resting cells Akt is sequestered in the cytoplasm. Upon stimulation Akt translocates to the membrane where it achieves full activation through phosphorylation at Ser and Thr [66]. In particular, 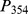 and 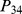 coordinate AKT activation by recruiting Akt and its kinase PDK-1 to the plasma membrane [67]. 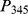 and 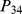, however, are not equivalent in this respect, as Akt binds 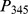 with slightly greater affinity than 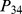, and Ser phosphorylation of Akt requires 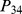 [67]. Although the majority of Akt targets are cytosolic, our focus is on the negative regulation of membrane proximal Raf by Akt. For this reason our model tracks the concentration of membrane bound Akt which we denote by 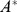. Because Akt activation is a complex process involving multiple steps of undetermined significance we resort to a simplified model, in which PI(345)P and PI(34)P are treated as equivalent and the activity of Akt at the membrane is approximated by the concentration of membrane bound Akt. Assuming that the concentration of unbound PI(345)P and PI(34)P is approximately equal to the total concentration of PI(345)P and PI(34)P, we derive the following equation for 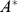 at steady state:
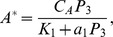
where 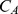 is the total concentration of Akt in the macrophage and, 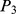 is the concentration of PI(345)P and PI(34)P at the immunological synapse, and 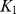 is the equilibrium dissociation constant of the Akt-PI(345)P complex. Given the rapid translocation of Akt to the membrane in response to 3PI production we approximate 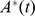 as
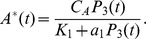
We set the parameter 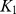 to be equal to the equilibrium dissociation constant for the Akt PH domain-PI(345)P complex which was measured in [68] through surface plasmon resonance. As we were unable to find a quantitative estimate of the cellular concentration of Akt in macrophages we take the cellular concentration of Akt, 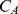, to be equal to that of PC12 cells, which was measured in [69].

### Rac and Ras

Rac and Ras are examples of GTPases which are proteins that cycle between an inactive GDP bound form and an active GTP bound form. GTPase activity is tightly regulated by guanosine nucleotide exchange factors (GEFs), GTPase activating proteins (GAPs), and in some cases guanosine dissociation inhibitors (GDIs) [70]. In resting cells GTPases are maintained in their inactive GDP bound form by their slow intrinsic rate of guanosine nucleotide dissociation. Upon stimulation, GEFs activate GTPases by accelerating the dissociation of GDP [70]. Furthermore, because the intrinsic rate of GTPases hydrolysis is also extremely slow, GTPase deactivation is mediated by GAPs that catalyze the reaction [70].

Rac is a GTPase of the Rho family that is activated by both CR3 and TLR2 regulates a variety of cellular processes including cytoskeleton rearrangements and cytokine production [30], [70], [71]. Multiple GAPs and GEFs contextualize Rac's response to stimuli. In PC12 cells, a positive feedback loop, involving Vav, Rac, and PI3K maintains Rac activity in response to NGF [72]. Indeed, studies indicate that PI(345)P enhances Vav's GEF activity by disrupting inhibitory intramolecular interactions [38]. Since PI(345)P production is required for CR3 mediated phagocytosis of *Francisella* [21], and Vav is responsible for Rho GTPase activation downstream of CR3 [71], it seems likely that a similar feedback loop is operative in this context. In addition to phosphoinositide binding, Vav is regulated through phosphorylation [73]. As the CR3 effector Lyn, can both phosphorylate Vav [74], and assist in the activation of PI3K [75] we propose a model in which Lyn induces a positive Vav/Rac/PI3K feedback loop. Meanwhile, in response to bacteria, TLR2 activates both Ras and Rac [28], [29], [76]. Although the precise mechanisms of activation are unknown Ras can be activated through direct association with TLR2 [29], and so we propose a model in which TLR2 activates Ras, which then activates Rac through the Rac GEF TIAM-1 [77]. These two modes of Rac activation are distinguished by their relation to PI3K. CR3-mediated Rac activation is PI3K-dependent, while TLR2-mediated Rac activation is PI3K-independent. In either case, however, both Rac and Ras are subject to PI3K-dependent deactivation, since both molecules are also regulated by PI3K sensitive GAPs [34], [35], [78].

Our model of Rac activity assumes that the concentrations of active Vav and GAP depend on the concentration of Lyn and PI(345)P as follows:
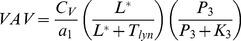
(9)

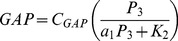
(10)


As PI(45)P has been shown to inhibit the GEF activity of Vav, we consider fully active Vav to be that which is both bound to PI(354)P and phosphorylated by Lyn. The fraction of Vav bound to PI(345)P is determined by 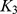 the equilibrium dissociation constant of the PI(345)P-Vav complex. Although precise measurements of the quantity were not available the equilibrium dissociation constant for the PI(45)P-Vav complex was measured in (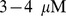), and the affinity of Vav for PI(345)P is known to be greater than the affinity of Vav for PI(45)P [79]. Hence we estimate 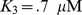. We were unable to determine the cellular concentration of Vav. We assume it is somewhat less than the concentration of Rac.

We were unable to determine the rates of Vav phosphorylation and dephosphorylation. As a result the fraction of phosphorylated Vav is determined by the unknown parameter 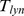 which is varied in the course of our numerical simulations. The concentration of Rac GAP at the membrane is determined by the equilibrium dissociation constant of the Gap-PI(345)P complex, 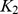. As we were unable to find a measurement of 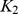 we choose this parameter so that the simulated time course of Rac activation during CR3-mediated phagocytosis would resemble the experimentally determined time course of Rac activation during Fc-Receptor-mediated phagocytosis [80]. Although PI(345)P stimulated GAPs are responsible for regulating the activity of Rac in macrophages, the cellular concentration of these GAPs was not available [81]. For this reason we assume that the concentration of PI(345)P responsive Rac-GAP is somewhat less than the concentration of Rac. For simplicity we treat TIAM-1 and Vav as equivalent Rac GEFs, and assume that the concentration of TIAM-1 is equal to the concentration of active Ras. This leads to the following model of Rac activation
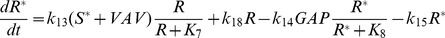
(11)

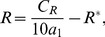
(12)where the constant 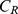 denotes the cellular concentration of Rac and 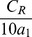 is the membrane proximal concentration of Rac under the assumption that all of the cellular Rac is concentrated at the membrane. The cellular concentration of Rac in neutrophils was measured in [82]. This is likely a good approximation to the actual concentration in macrophages. The rate parameters 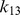 and 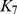 characterize TIAM-1 catalyzed nucleotide dissociation from Rac2 as determined in [83]. The intrinsic rate of Rac2 hydrolysis 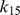 and the rate of spontaneous GDP dissociation 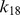 were measured in the same work. We were unable to find measurements for the parameters 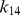 and 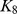 that characterize the GAP-catalyzed hydrolysis of GTP by Rac, and so, we estimate these parameters using the parameters from the 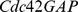-catalyzed hydrolysis of GTP by Cdc42 [84]. This is a reasonable approximation since Cdc42 is closely related to Rac.

Ras is a GTPase that mediates ERK activation by recruiting Raf to the plasma membrane [30]. Since the mechanism through which TLR2 activates Ras is unknown we treat TLR2 as a Ras GEF.
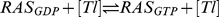
We model the GAP induced deactivation of Ras as an enzymatic process in which GAP catalyzes the hydrolysis of GTP to GDP.
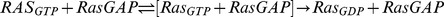



Combing these two processes, i.e. the exchange of GDP for GTP and the hydrolysis of GTP to GDP, we obtain the following equation for Ras.
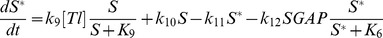
(13)

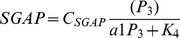
(14)

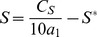
(15)where the constant 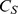 denotes the total cellular concentration of Ras, and 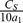 is the membrane proximal concentration of Ras under the assumption that all of the cellular Ras is concentrated at the membrane. We take the cellular concentration of Ras in macrophages to be equal to that in NIH3T3 fibroblasts [85]. The parameter 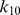 represents spontaneous dissociation of Ras-GDP. This parameter was determined in vitro [86]. The parameters 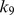 and 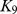 which determine the kinetics of Ras activation by TLR2 are not known. We take these parameters from a study on the kinetics of Ras activation by the exchange factor Cdc25 [87]. The parameters, 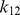, and 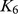, which catalyze GAP catalyzed hydrolysis were measured in [88]. The equilibrium dissociation constant, 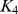, between the Ras GAP and PI(345)P was reported in [37]. Although PI(345)P sensitive Ras-GAPs are expressed by macrophages [89], [90], we were unable to find estimates of their levels of expression in macrophages. Hence, we assume that the concentration of Ras-GAP is somewhat less than the concentration of Ras.

### Phosphoinositides

Phosphatidylinositol, or PtdIns, is a membrane lipid that mediates signal transduction between cell surface receptors and the cytosol. Its inositol head group contains several free hydroxyls which can be phosphorylated to generate a variety of distinct derivatives termed phosphoinositides [91]. For brevity we abbreviate PtdIns by PI and the phosphoinositides by PI()P, where the terms in the parentheses correspond to the phosphates' positions. For example PtdIns(4,5)P is abbreviated as PI(45)P. Distinct phosphoinositides transduce distinct signals depending on the location and number of phosphates they contain. In particular, several pivotal proteins bind to their partner phophsoinositides with high specificity [92], [93]. This allows phosphoinositides to determine the activity of these proteins, by localizing them to cell membranes. In several instances, phosphoinositides can also serve as allosteric activators.

In vivo the inositol head group of PtdIns can be phosphorylated at positions 3, 4, and 5 [91]. The membranes of resting cells contain minute quantities of PI(4)P and PI(45)P, while PI(345)P andPI(34)P are virtually undetectable [92], [93]. Upon stimulation, however, a variety of cell surface receptors, including CR3, induce a rapid increase in the concentration of the 3 phosphoinositides which are important mediators of cytokine production, phagocytosis and chemotaxis [91], [94]. In particular, the generation of 3 phosphoinositides is an essential step in the phagocytosis of bacteria [95], [96].

Experiments conducted in vivo suggest that phosphoinositide production proceeds according to the following diagram [97]:
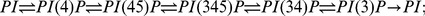
in particular, hydrolysis of PI(345)P is the primary mode of PI(34)P production. Stimulation of TLR2 and CR3 is known to induce phosphoinositide 3 and phosphoinositide 5 kinases as well as phosphoinositide 5 phosphatases. The Src kinase Lyn and the small GTPase Rac both contribute to the activation of PI3K. Rac-GTP contributes to PI3K activation by binding to its regulatory subunit [29], while Lyn activates PI3K through Cbl [31], [32]. As the parameters with which Lyn and Rac activate PI3K are not known, we take the concentration of active PI3K to be a function of the concentration of active Lyn and Rac,
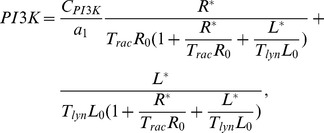
(16)where 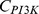 is the cellular concentration of PI3K, which we assume to be a constant, and 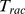, 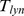 are the factors by which the levels of active Rac and Lyn must be elevated over their basal values, 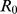 and 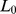 respectively, in order to support half maximal activation of 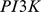. For simplicity we will assume that the two species of 3 phosphoinositides PI(34)P and PI(345)P are equivalent and we will neglect PI, PI(4)P, and PI(3)P so that in the model PI(34)P and PI(345)P are synthesized directly from PI(45)P and PI(45)P is the direct product of PI(345)P and PI(34)P degradation. As this part of our model is largely conceptual the values of the parameters 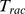 and 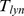 are unknown and are varied in the course of the numerical simulations. The cellular concentration of PI3K, 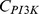, is taken from a study NIH3T3 fibroblasts [69]. The basal activity of Rac and Lyn, 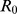 and 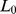 is determined by the model. With these assumptions, the production and degradation of the PI(34)P and PI(345)P are described by the following equation,
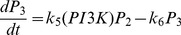
(17)

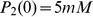
(18)

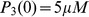
(19)

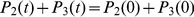
(20)where 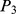 denotes the concentration of PI(34)P and PI(345)P and 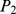 denotes the concentration of PI(45)P, and the total concentration of PI(45)P, PI(34)P, and PI(345)P is assumed to be a constant.

The concentration of PI(45)P, PI(345)P and PI(34)P in resting neutrophils [97] was used to determine the initial conditions 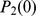 and 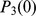. We were unable to find estimates of the parameters 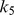 and 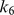 which determine the kinetics of CR3-stimulated PI(345)P formation in macrophages. A study of PI(345)P formation during Fc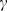 Receptor-mediated phagocytosis in macrophages reported rapid and substantial accumulation of PI(345)P, with maximal levels reached 30–90 seconds after stimulation [96]. Furthermore the rate of PI(345)P degradation during this process, 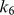, was estimated to be somewhat greater than 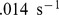. Initial conditions (18)–(19) along with (20) and 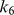 enable us to estimate 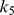.

### Raf

In its inactive state Raf is sequestered by 14-3-3 binding proteins, the association of which is supported by phosphorylation at Ser 259 [98], [99]. Dephosphorylation of Raf at Ser 259 precedes Raf activation. Similarly, phosphorylation of Raf at Ser 259 precedes Raf deactivation [98], [99]. Although the kinase responsible for phosphorylating Ser 259 in this context is unknown, Akt has been demonstrated to regulate Raf through phosphorylation at Ser 259 in several systems [33], [98], and so, we assume that this is the case. Activation of Raf is a multistep process. Raf is recruited to the membrane by Ras-GTP, to which it binds with high affinity. This recruitment places Raf in close proximity to kinases that activate Raf through phosphorylation [100]. One such kinase, Pak, is activated after binding to active Rac [30] The Src family kinase Lyn can also activate Raf [39]. Because many of the parameters that describe the transition between these many states of Raf are unknown we consider a simple model in which Raf exists in three states: active membrane proximal Raf, 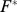, inactive and free Raf, 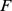, and inactive Raf that is bound to Ras. For simplicity we assume that recruitment of Raf by Ras-GTP is rapid, so that a the fraction of inactive Ras-bound Raf is
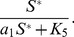
(21)The equilibrium dissociation constant for the Raf RBD-Ras complex, 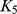, was measured in [100]. In standard Michaelis-Menton kinetics, the substrate concentration is generally assumed to be in excess of the enzyme concentration. Because cellular concentrations of Raf are extremely low, this assumption is unlikely to hold for Raf. Hence in our model of Raf activation and deactivation we employ a modified Michaelis-Menton type model in which Raf is limiting so that the reaction rates depend linearly on the concentration of Raf. Furthermore, since the differences between Lyn- and Pak-catalyzed phosphorylation of Raf are unknown we assume that the concentration of active Pak is proportional to the concentration of active Rac, and that Pak and Lyn are equivalent. With these assumptions, the concentration of active Raf is determined by the following equation.
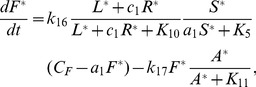
(22)where 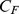 denotes the cellular concentration of Raf, which we assume is a constant, and 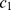 determines the fraction of Pak that is bound to Rac and therefore active. We were unable to find the concentration of Raf in macrophages or closely related cells and have estimated 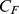 from data on COS cells [69]. The parameter 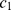 is unknown. We set its baseline value to one and vary it in the course of numerical experiments. We were unable to find the specific parameters 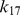 and 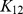 that determine the kinetics with which Akt phosphorylates Raf in macrophages. Instead we estimate these parameters to be equal to the parameters that determine the kinetics of the reaction between Akt and a small peptide substrate [101] under the assumptions that the concentration of ATP in a resting pig alveolar macrophage is equal to that of resting human alveolar macrophage [102], and that the volume of a pig alveolar macrophage is 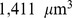 [103]. The parameters which determine the rate of Raf activation by Lyn and Pak are also unknown. We estimate them from knowledge of the kinetics with which closely related Src family kinases catalyze the phosphorylation of their substrates [104], [105], and the kinetics with which Raf is phosphorylated on the plasma membrane of living cells [106].

We should note that Raf is also subject to ERK-mediated negative feedback. Indeed ERK was shown to inhibit Raf kinase activity by phosphorylating Raf at several sites [40]. Although this feedback plays an important role in determining the duration of Raf signaling, it does not significantly influence the activity of Raf at early time points [40]. Since our intent is only to describe the very earliest of signaling events, our model neglects this feedback.
